# Application Options of the Sustainable Child Development Index (SCDI)—Assessing the Status of Sustainable Development and Establishing Social Impact Pathways

**DOI:** 10.3390/ijerph15071391

**Published:** 2018-07-02

**Authors:** Ya-Ju Chang, Annekatrin Lehmann, Lisa Winter, Matthias Finkbeiner

**Affiliations:** Institute of Environmental Technology, Technische Universität Berlin, Straße des 17. Juni 135, 10623 Berlin, Germany; annekatrin.lehmann@tu-berlin.de (A.L.); lisa.winter@campus.tu-berlin.de (L.W.); matthias.finkbeiner@tu-berlin.de (M.F.)

**Keywords:** Sustainable Child Development Index (SCDI), sustainable assessment, sustainable development, child development, Social Life Cycle Assessment (SLCA), social impact pathways, tertiary education, poverty, Social Organizational Life Cycle Assessment (SOLCA), The Social Hotspots Database (SHDB)

## Abstract

The needs of children and their vulnerability to diseases, violence and poverty are different from those of adults. The Sustainable Child Development Index (SCDI) was thus developed in previous work to evaluate the status of sustainable development for countries with a focus on children and triple-bottom-line thinking. This study proposes application options to put the SCDI into practice. The SCDI can be performed similarly to existing development indices, for comparing and tracing the performance of sustainable development on different geographic levels and between population groups. In addition, the SCDI can be integrated into existing social sustainability assessment approaches (e.g., Social Life Cycle Assessment and Social Organizational Life Cycle Assessment) and databases (e.g., The Social Hotspots Database) to take children into account and enhance impact assessment of social sustainability assessment approaches. As an exemplification, this study demonstrates the application of the SCDI framework to support the development of social impact pathways. Due to the importance of tertiary education in reducing poverty, a preliminary social impact pathway addressing completion of tertiary education was established. By putting the SCDI into practice, the SCDI can support decision making in child as well as sustainable development policies.

## 1. Introduction

Children (defined as aged under 18 according to the United Nations [1]) are key stakeholder for achieving sustainable development because they inherit and shape societies. According to the Brundtland Report [2], sustainable development is defined as “a development that meets the needs of the present without compromising the ability of future generations to meet their own needs”. This definition stresses intra- and inter-generational equity and denotes that every adult and child have the right to own the opportunity to develop in freedom and in a stabilized society by satisfying basic needs and protecting the environment. In addition, children are more vulnerable than adults to diseases, environmental pollution, violence and poverty, and their specific needs are different from those of adults. Overlooking negative living conditions (e.g., poverty and violence) in childhood can compromise life experience for children and impede their long-term development. Due to these reasons, an index for evaluating sustainable development with a focus on children is needed to complement existing whole-population-oriented assessments, such as the Human Development Index (HDI) [3]. For instance, the HDI was established to present the development status of a country by aggregating indicators for health, knowledge, and standard of living in accordance with national average data with regard to whole population. It has been widely applied for decades, but future generations, i.e., children, are not considered.

Some development indices have been proposed with a focus on children, but they address social and economic indicators and have not yet considered other indicators regarding sustainable development, such as environmental aspects. For example, the Child Development Index (CDI) was proposed to evaluate the development of children considering health (i.e., under-five mortality), education (i.e., primary school enrolment) and nutrition (i.e., underweight), without considering issues associated with environmental aspects [4]. Among the issues of environmental aspects, resource accessibility is of significance that ensures future generations to live with accessible and abundant resources. For example, availability of freshwater and preservation of fossil fuels are of high importance to reflect resource scarcity and shall be considered into development indices and sustainability assessments.

Therefore, the Sustainable Child Development Index (SCDI), which considers children as a core and addresses children related topics in the context of sustainable development, was established in previous work for supporting decision making in child development and sustainable development policies [5,6,7]. The SCDI allows for comparing countries in terms of their status regarding sustainable child development and for monitoring the performance of countries by updating the indicators over time. Sustainable child development refers to a development that supports children to meet their needs in the present living state and protects children to have ability for shaping their future prospects. Compared to sustainable development defined by Brundtland Report, sustainable child development takes children as a core and particularly stresses that the children should be supported and protected to satisfy their needs and to be capable to develop themselves during both current and future stages. Hence, the SCDI not only evaluates the current development status (e.g., eliminating risk behavior and reducing mortality) but also considers the restrictions that limit future development of children (e.g., scarcity of nature resources).

**Figure 1:**
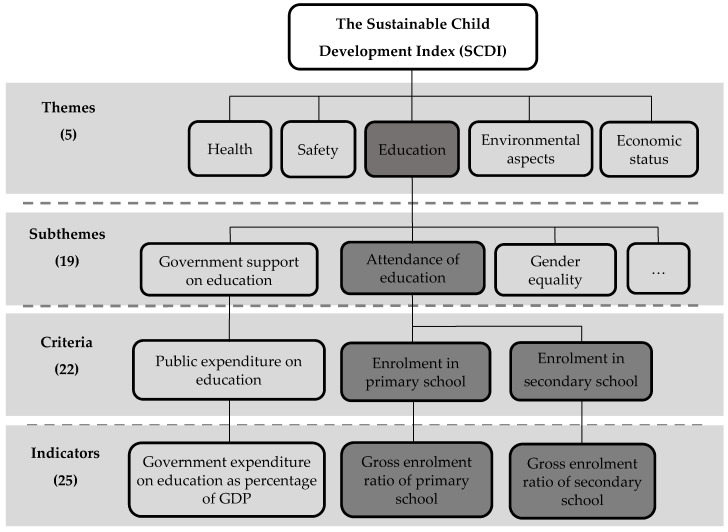
The SCDI framework (exemplary criteria and indicators are presented for the subtheme attendance of education belonging to the theme education and highlighted in dark grey), adapted from Chang et al. [5,6,7].

Previous work of the authors focused on the construction of the SCDI and the comparison of the SCDI and other existing development indices. First, topics, indicators and gaps associated with evaluating sustainable child development were identified to propose the framework of the SCDI [5,6]. As shown in Figure 1, the identified relevant topics were then classified into a hierarchical framework, consisting of themes, subthemes and criteria. Each theme (e.g., education) is specified by subthemes (e.g., attendance of education and government support on education), which are further described by criteria (e.g., enrolment in primary school and public expenditure on education), measured by indicators (e.g., gross enrolment rate of primary school and government expenditure on education as percentage of GDP) [5,6]. An indicator set was then selected regarding: (1) data availability, (2) association between indicators and (3) coverage of considered subthemes, criteria, countries and child population in the SCDI [6,7]. On the basis of the indicator set, the SCDI at present addresses five themes (health, education, safety, economic status and environmental aspects) described by 19 corresponding subthemes (e.g., child mortality, risk behavior, gender equality in education, macroeconomic situation and renewable energy consumption) measured by 25 indicators. The numbers in brackets in Figure 1 display the numbers of themes, subthemes, criteria and indicators considered in the indicator set. So far, the SCDI was calculated for 138 countries [7]. As being internationally agreed targets for sustainable development, the Sustainable Development Goals (SDGs) [8] were used to define the reference points for the indicators to evaluate countries’ status of sustainable child development. The SCDI was designed as an aggregated score ranging between 0–1. The higher the SCDI score is, the better is the sustainable child development status for a country [7]. For example, the SCDI score for Sweden and Argentina is 0.840 and 0.710, respectively, indicating that Sweden has a better sustainable child development status than Argentina. Chang et al. [7] also demonstrated that the SCDI complements existing development indices (e.g., the HDI and the CDI) to support a more comprehensive evaluation of sustainable development for countries. That is, the SCDI can evaluate the sustainable development status for countries differently than the HDI and the CDI by treating children as key stakeholders and by addressing environmental and additional topics (e.g., safety) connected to sustainable development.

Though the SCDI was established and its ability to complement existing sustainable development indices was demonstrated in previous work, it has not yet been applied in practice within sustainability assessments. Thus, the objective of this study is to propose application options of the SCDI. The application options are proposed by taking current practices of sustainability assessments (e.g., development indices and social sustainability assessment approaches and databases) into account. In this study, two potential ways to implement the SCDI for supporting decision making in development policies and enhancing existing sustainability assessments are proposed by the authors:

- Applying the SCDI similarly to the current practice of existing development indices (e.g., the HDI [3]) to assess the performance of sustainable development on different geographic levels and between population groups
- Integrating the SCDI framework into social sustainability assessment approaches (e.g., Social Life Cycle Assessment, SLCA [9] and Social Organizational Life Cycle Assessment, SOLCA [10]) and databases (e.g., Social Hotspots Database, SHDB [11]) to consider children as a stakeholder group and to enhance social impact assessment

The following sections present the methodology (Section 2), the recommended application options of the SCDI—similar to the current practice of existing development indices (Section 3) and integration of the SCDI framework into existing social sustainability assessment approaches and databases (Section 4) as well as discussion (Section 5). 

## 2. Methodology

This section presents, how application options of the SCDI were identified and selected. To identify potential application options, the current practice of existing development indices (Section 2.1) and social sustainability assessment approaches and databases (Section 2.2) were reviewed. Application options proposed by the authors and corresponding examples are provided in Section 3 and Section 4 afterwards.

### 2.1. Reviewing the Current Practices of Existing Development Indices

Chang et al. [7] demonstrated that the SCDI complements the HDI. Therefore, current practices of the HDI were taken as references to suggest application options of the SCDI. The HDI was introduced by the United Nations Development Programme (UNDP) in the 1990s [12] and has been widely adopted in development studies and policy making to measure the development status of a country based on national average data of the whole population. Practices of the HDI in development studies and policy making were first reviewed by the authors. Based on this literature review, three types of practices of the HDI were identified: evaluating status of sustainable development: (1) on different geographic levels and (2) between population groups, and (3) for establishing other development indices.

### 2.2. Reviewing Existing Social Sustainability Assessment Approaches and Databases

As the SCDI addresses social and economic topics which are also assessed in existing child development as well as sustainable development research, the integration of the SCDI framework into current social sustainability assessment approaches and databases was considered as a potential application option. Social sustainability assessment approaches (Section 2.2.1) and databases (Section 2.2.2) were reviewed to investigate application options of the SCDI.

#### 2.2.1. Social Sustainability Assessment Approaches

Building upon life-cycle based social sustainability assessment approaches [10,13,14,15,16,17], the authors proposed to apply the SCDI framework within Social Life Cycle Assessment (SLCA) and Social Organizational Life Cycle Assessment (SOLCA). In this Section, the background of SLCA and SOLCA is first introduced to facilitate understanding. Then, the approaches to demonstrate the integration of the SCDI framework into SLCA and SOLCA are explained.

SLCA was established in 2009 [9] and assesses social and socio-economic impacts of products from a life cycle perspective. According to The Guidelines for SLCA of products (hereafter referred to the Guidelines), social and socio-economic impacts can affect different stakeholder groups: workers, consumers, local communities, value chain actors and the society [9]. The SLCA framework defined in the Guidelines builds upon relevant socio-economic topics called subcategories which are measured by so-called inventory indicators. Subcategories can be aggregated to stakeholder groups and/or to impact categories. For example, for the stakeholder group workers, eight subcategories (e.g., freedom of association and collective bargaining, fair salary, and working hours) are suggested. The aggregation of subcategories into impact categories can help to integrate the results of the subcategories that have the same impacts [9]. The aggregated results could be further linked to areas of protection, e.g., human well-being. Abiding by the Guidelines, the ‘Methodological Sheets for Subcategories in SLCA’ (hereafter referred to the Methodological Sheets) were published to provide a practical guidance on how to evaluate the subcategories by suggesting indicators for its measurements [18]. Subcategories and indicators considered in the Methodological Sheets are specified for different stakeholder groups.

SOLCA was developed in 2015 and adapts the SLCA framework to an organizational level for providing a more direct evaluation of social and socio-economic impacts resulted from organizational behavior and context [10]. As most social impacts addressed in SLCA are influenced by organization’s behavior and national conditions (e.g., fair salary) rather than a product, an organizational approach may be more straightforward than a product approach to address social aspects. The conceptual framework of SOLCA was built based on the Guidelines and the Guidance on Organizational Life Cycle Assessment (OLCA) [19]—Which adapts product LCA to the organizational perspective. SOLCA inherits the considered stakeholder groups, subcategories and impact categories from the SLCA framework. Impact assessment and interpretation of SOLCA are mostly mapped according to SLCA [10]. Table 1 summarizes the key differences between the SLCA and SOLCA framework, e.g., different goal and unit of analysis.

So far, the missing consideration of children as a stakeholder group and a low development of quantitative social impact assessment method have been two of the challenges of SLCA and SOLCA. Though children are relevant in supporting sustainable development, children have not yet been considered as a stakeholder group in SLCA and SOLCA. For example, the Guidelines name future generations as a stakeholder group which can be optionally considered in SLCA studies. The Methodological Sheets do not suggest a corresponding framework and indicators for taking future generations into assessment [9]. Child labor is the only children-related subcategory in the Guidelines. Such a low concern on children’s interests and their influence on sustainable development may consequently lead to a biased interpretation of social sustainability.

Lacking quantitative social impact pathways that describe relation between socio-economic topics is another challenge of SLCA and SOLCA [10,13]. Impact assessment aims at relating indicators for socio-economic topics to real impacts. Most of the SLCA studies (e.g., Ekener-Petersen & Finnveden [20] and Martínez-Blanco et al. [16]) applied performance reference points to conduct impact assessment for qualitatively or semi-quantitatively indicating the levels of social performance or impacts. The interwoven connection between socio-economic topics and the common usage of qualitative indicators brings the difficulty to describe the relation of socio-economic topics in a quantitative way. This difficulty consequently hinders the implementation of SLCA and SOLCA, and lead to an incomplete consideration of potential social impacts in SLCA and SOLCA studies.

Since the SCDI considers children as a stakeholder group and its framework encompasses and classifies the relevant topics of sustainable child development, this study investigates how the SCDI framework could be used to address the two challenges of SLCA and SOLCA.

First, as the SCDI is a children-oriented assessment, the SCDI framework could be used to suggest impact categories, subcategories and indicators which shall be addressed for a newly proposed stakeholder group, i.e., children, in SLCA and SOLCA. Furthermore, the SCDI could be directly used as a stakeholder-oriented index to assess the social conditions specified for the proposed stakeholder group children and to initiate the development of other stakeholder-oriented indices for other stakeholder groups in SLCA and SOLCA.

Second, the SCDI framework could be a laying ground to initiate the development of social impact pathways. The SCDI framework provides qualitative description of the relation between the themes, subthemes and criteria (see Figure 1). To have a closer investigation of the relation between the SCDI criteria, this study examined the relation between the selected SCDI criteria from different themes and provided quantitative description of the relation. When the interlinkages between the SCDI criteria across different themes are demonstrated, the results can in turn support the interwoven nature of socio-economic topics. For example, education can relate to health or safety topics. According to the examined relation between the selected SCDI criteria, a preliminary social impact pathway could be proposed.

Path analysis was applied to examine the validity of relation and to quantify the strength of relation between selected criteria. Path analysis is a statistical technique extending from linear regression that can examine if a given data set fits the hypothesized relations specified in the hypothesized relation model and can assess the strength of relation between the selected criteria along hypothesized pathways [21]. This technique was firstly proposed by Sewall Wright [22,23] and has been applied in phylogenetic, social and behavioral studies [24,25,26,27,28,29].

Path analysis consists of three steps. First, a relation model (hereafter referred to exemplary socio-economic relation model) was established to consider the hypothesized relations between criteria. Second, linear regression was performed to examine the validity of hypothesized relations. Path coefficients from linear regression present relative magnitude and the sign (positive or negative) of the relation between criteria. The value of the path coefficient varies between +1 and −1. Path coefficient of ±1 occurs if a criterion could potentially contribute all the (positive or negative) attribution to another criterion. Therefore, this study uses path coefficients to compare the magnitude of the relation between criteria. Third, direct and indirect relation between criteria was investigated according to the results of linear regression. The strength of indirect relation between two criteria was estimated by multiplying the path coefficients along the pathways between the two criteria [21,25,27,28].

An exemplary socio-economic relation model was thus established for showing how to use the SCDI framework and path analysis to initiate the development of social impact pathways. Tertiary education (referring to both public and private universities, colleges, advanced vocational and professional education [30]) is significant in diminishing poverty and fostering growth. Verner [31] found that completed tertiary education reduces poverty more effectively than secondary education. Also, population who complete tertiary education are six times less likely to fall below the poverty line than those who complete primary education. The economic return for tertiary education graduates is estimated 17% enlargement in earnings as compared with 10% for primary and 7% for secondary education [32]. As tertiary education is a key to tackle poverty, the relation between the criterion completion of tertiary education and other criteria selected from the SCDI framework was analyzed in the exemplification.

The results of correlation analysis performed in Chang et al. [7] was used to choose the criteria associated with the criterion completion of tertiary education. Six criteria of the SCDI were chosen as their corresponding indicators were found to have association with the indicator for the criterion completion of tertiary education: enrolment in tertiary education, children involved in child labor, children married or in union, adolescent fertility, public expenditure on tertiary education, and household and ambient air pollution. Then, it is reasonable to further examine and quantify the relation between the six selected criteria and the criterion completion of tertiary education. The criteria completion of tertiary education, enrolment in tertiary education and public expenditure on tertiary education are classified into the theme education in the SCDI framework. The criteria children involved in child labor, and children married or in union are categorized into the theme safety; the criteria adolescent fertility and household and ambient air pollution are selected from the theme health. Spreadsheet S1 in the Supplementary Material provides the seven indicators and their latest statistical data used in the path analysis. The numbers of addressed countries and the reference year of the statistical data for the indicators are also presented in Spreadsheet S1. Spreadsheet S2 in the Supplementary Material provides the results of correlation analysis for the seven considered indicators. Hypotheses of the relations between the six selected criteria and the criterion completion of tertiary education were defined by the authors based on the correlation analysis and are described in Spreadsheet S3 in the Supplementary Material. The path analysis was then programmed by IBM SPSS Statistics [33]. It should be noticed that the exemplary socio-economic relation model only considered some of the possible relations to demonstrate the quantification of relation between criteria.

The causality between the selected criteria is hard to examine and quantify due to the interconnection of socio-economic topics and the difficulty in determining the causality within temporal sequence. Therefore, this study focuses on a quantitative assessment of the relation of the selected criteria to get a step closer to the causality of socio-economic topics.

#### 2.2.2. Social Sustainability Databases

Integrating the SCDI into a social sustainability database can support the generic assessment of social condition and development status for countries. According to the experience of using social sustainability databases [34], the authors selected and examined the Social Hotspots Database (SHDB). In current social sustainability studies, the SHDB is usually used a screening tool to provide a generic assessment by identifying the social hotspots for countries and sectors [11,35]. The SHDB has been developed by New Earth and provides social risk information on five categories, namely human rights, health and safety, labor rights and decent work, governance, and community infrastructure, described by 22 social themes including 89 issues characterized for risk for countries or country-specific-sectors and 133 indicators [11,35,36]. Practitioners can thus compare and analyze risks for individual social issues for countries or selected country-specific-sectors (e.g., German manufacturing sector) in a supply chain. The Social Hotspots Index (SHI), which considers topics and indicators selected from the SHDB, is available to summarize the large amount of social risk information for the country-specific-sectors in a supply chain [11,35]. The SHI considers 39 indicators for the five categories and 22 themes selected, and is determined by means of a weighted sum approach [37]. However, identical to the challenge identified in SLCA and SOLCA, there is a limited consideration of children in the SHDB. For example, only three out of the 133 indicators used in the SHDB address health, education and child labor issues connected to sustainable child development. Among the 39 indicators considered in the SHI, only two indicators (i.e., percentage of child labor and percentage of children out of primary education) are directly linked to children. Hence, using the SCDI to tackle this challenge was also taken into consideration to propose application options of the SCDI. A case study involving a bamboo bike supply chain [34] was conducted to compare the social condition assessed by the SCDI and the SHI.

## 3. Application of the SCDI Similar to Existing Development Indices

According to Chang et al. [7], the SCDI enables a complementary assessment to whole-population oriented indices, such as Human Development Index (HDI), by assessing sustainable development performance for countries or regions with a focus on children. Three applications for the SCDI based on the present use of the HDI are suggested as follows: (1) evaluating the achievement of sustainable child development on different geographic levels, (2) comparing the development condition between population groups, and (3) being as basis to establish further development indices.

First, the SCDI can be used to assess the status of sustainable child development on different geographic levels. Basically, the SCDI can be used to compare and monitor countries’ achievement regarding sustainable child development. Like the HDI results presented in the annual human development reports published by the United Nations Development Programme [38], the trends on enhancements and declines of the performance for countries regarding sustainable child development can be continually updated. The SCDI results can thus provide information for both the sustainable child development status, and support policy making by showing hotspots of the considered topics of sustainable child development. Regarding the indicators with different update frequency, an updating frequency of the SCDI is suggested as 1–4 years. This proposed updating frequency could be reasonable and realistic since a longer time frame (than just one year) may be required to clearly reflect the trend of the country’s performance [6,7].

Furthermore, breaking down from country level, the SCDI can analyze the inequality on the sustainable child development between regions and cities for supporting policy making. Some existing studies which applied the HDI on regional level can serve as basis to suggest applications of the SCDI. For example, Schrott et al. [39] modified the HDI to assess the development status across the provinces of Austria. The results showed substantial differences of the HDI results in life expectancy between the provinces. The study also found an inequality of income and educational level within- and between provinces, emphasizing the needs of policies to lower the infrastructure weakness in rural regions. Antony and Visweswara Rao [40] used both the HDI and Human Poverty Index to analyze the variations in poverty, health, nutritional status and standard of living among Indian states, and concluded that demographic, socio-economic, health and dietary indicators determined the real standard of living for India.

Moreover, the SCDI can be performed similarly to the HDI to describe the development condition between population groups (e.g., different ethnic and income groups). For instance, Segura and Birson [41] adapted the HDI and found an inequality in human capital and social well-being between the ethnic groups within the United States. The findings revealed the needs for addressing the gaps in the unequal development status between the ethnic groups. Cooke et al. [42] applied the HDI to compare the development of indigenous people and the general populations in Australia, Canada, New Zealand and the United States. The assessed countries were evaluated as high human development countries, nevertheless, their resident indigenous people were only recognized in medium human development, calling for the efforts to improve the living condition of indigenous people. Especially, Australian society faced the increasing gap in human development between indigenous people and the general population. The results indicated that the Australian government shall address this development gap in the existing development policy in priority. Focusing on the socio-economic distribution, Harttgen and Klasen [43] and Grimm et al. [44] performed the HDI at the household level to capture the inequality of human development between income groups within and among countries. The studies proved that inequality was large for countries assessed as low human development countries, especially for African countries. The results could raise awareness to governments to take measures to tackle income inequality for lowering the gap in development progress within their countries. By following those applications, the SCDI allows for analyzing sustainable development achievement with a focus on children to explore the inequality on development achievement among population groups, such as different ethnics, income groups and education level, etc.

In addition, the SCDI can be used as basis for establishing further development indices. By including additional topics with regard to sustainable development e.g., poverty, the SCDI can be adapted and then applied for different specific assessment purposes. The indices derived from the HDI can be used as a reference to construct new indices based on the SCDI. For example, the HDI has been further adapted in many studies by considering specific topics associated to sustainable development such as inequality (e.g., Inequality-adjusted HDI [45]), deprivations level (e.g., Multidimensional Poverty Index [46]), and environment (Sustainability Adjusted HDI [47]). These indices derived from the HDI take the three dimensions of the HDI (i.e., long and healthy life, knowledge and a standard of living) as the core of schemes, and then the schemes are modified by considering additional topics. Via adding topics connected to sustainable development into the SCDI framework, these SCDI-derived indices can evaluate the status of sustainable child development for different purposes and can thus perform as tools to support decision-making in sustainable development policies with a focus on children.

## 4. Integration of the SCDI into Existing Social Sustainability Assessment Approaches and Databases

In this section, the integration of the SCDI framework into Social Life Cycle Assessment (SLCA) and Social Life Cycle Assessment (SOLCA) (Section 4.1), and the Social Hotspots Database (SHDB) (Section 4.2) is presented.

### 4.1. Integration of the SCDI Framework into SLCA and SOLCA Scheme

Two options of how the SCDI framework could be integrated into both the SLCA and SOLCA scheme are suggested and described in the following two subsections:

- Proposing children as a new regularly considered stakeholder group and suggesting its corresponding indicators, subcategories and impact categories (Section 4.1.1)
- Using the SCDI framework (e.g., criteria and indicators) for initiating the development of social impact pathways (Section 4.1.2)

#### 4.1.1. Proposal of Children as a New Stakeholder Group

**Figure 2:**
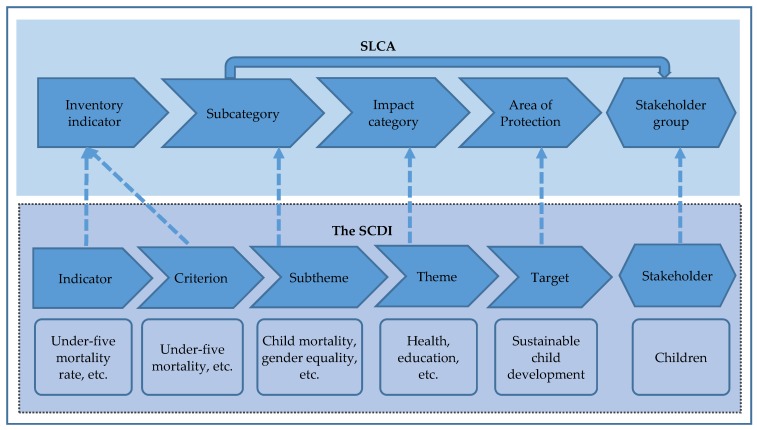
Scheme of SLCA and SOLCA (based on UNEP [9]) and an exemplary relation of the SCDI framework to SLCA and SOLCA.

The SCDI framework can complement the existing SLCA and SOLCA framework by laying a ground for establishing a new stakeholder group namely children and its according impact categories, subcategories and indicators. Children, inter-generational equity and sustainable development are inseparable from each other. In line with this concept, children shall thus be proposed as a regularly considered stakeholder group to ensure that sustainable development is considered in SLCA and SOLCA studies. The SCDI takes children as the core of assessment and consists of five themes (i.e., health, education, safety, economic status, and environmental aspects) relevant to sustainable child development. As shown in Figure 1 and Figure 2, each theme of the SCDI is specified by subthemes, and these subthemes are further described by criteria measured by indicators [5,6,7]. 

This hierarchical structure is similar to the SLCA framework: The five themes of the SCDI can be treated as impact categories in SLCA and SOLCA, and the subthemes of the SCDI can be applied as the subcategories for the newly proposed stakeholder group, children (see dotted lines in Figure 2). The five themes of the SCDI can be used as impact categories to aggregate the results of subcategories on a higher level in SLCA and SOLCA scheme. The criteria of the SCDI can be used to specify the associated subcategories in the SLCA and SOLCA framework and then assessed by the indicators. The impact categories, subcategories and indicators (adapted from the present SCDI framework [7]) proposed for the stakeholder group children are listed in Table 2. It shall be noticed that the assessment scope of the SCDI is not consistent with the assessment scope of SLCA and SOLCA. Compared to SLCA and SOLCA, the SCDI provides a broader assessment scope by further addressing environmental aspects.

New methodological sheets that contain the suggested subcategories and corresponding indicators (in Table 2) for the newly proposed stakeholder group (i.e., children) shall then be developed and complement the existing ones for the other stakeholder groups. It shall be noticed that so far there are no available studies providing the subcategories and corresponding indicators for stakeholder groups for SOLCA. Since SOLCA inherits the structure consisting of stakeholder groups and subcategories from SLCA, the methodological sheets that address the subcategories and corresponding indicators for children is suitable to SOLCA as well.

Moreover, the SCDI can be used as a stakeholder-oriented index to evaluate the social conditions specified for the stakeholder group children and initiate the development of other stakeholder-oriented indices for other stakeholder groups in SLCA and SOLCA. Since the SCDI scores are determined by combining several topics and indicators of sustainable child development, the SCDI can be used as an index to describe the social conditions for the stakeholder group children for countries in SLCA and SOLCA studies. This SCDI application can be an illustration for initiating stakeholder-oriented indices for other stakeholder groups in SLCA and SOLCA. For example, a stakeholder-oriented index for workers can be established by considering the subcategories connected to workers (e.g., the subcategories freedom of association and collective bargaining, fair salary, hours of work, and health and safety) for providing an overall assessment of social impacts on workers. This stakeholder-oriented index establishment can facilitate stakeholder-oriented analysis of social conditions in SLCA and SOLCA studies. This application is not identical to the first proposed application option which uses the SCDI compares and traces the status of sustainable child development on different geographic levels and between population groups (see Section 3). This application specifies how to use the SCDI to support stakeholder-oriented assessment in SLCA and SOLCA studies. Organizational behavior can be directly, significantly influenced by social conditions in different countries. At present the SCDI can evaluate an overall status of sustainable development as well as social conditions on country level. Therefore, compared to using the SCDI to link the generic social conditions to a specific product assessed in SLCA, the SCDI can provide a closer investigation of social context for an organization in SOLCA studies.

#### 4.1.2. Supporting the Development of Quantitative Social Impact Pathways

This section presents how to use the SCDI framework (e.g., themes, subthemes, criteria and indicators) as basis to initiate the development of social impact pathways for the impact assessment in SLCA and SOLCA. The results of path analysis showed the validity of the hypothesized relation between the selected criteria and provided quantitative information on the strength of the valid relation (Section 4.1.2.1). A preliminary social impact pathway was then established according to the results of path analysis (Section 4.1.2.2).

##### 4.1.2.1. Results of Path Analysis

**Figure 3:**
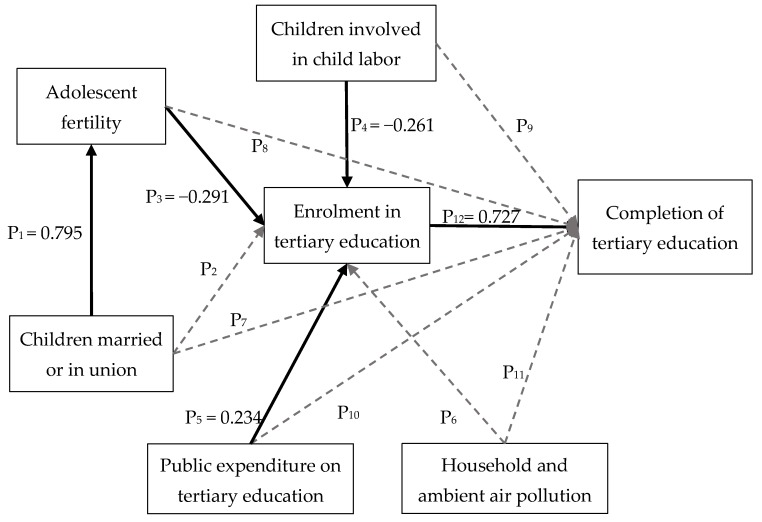
An exemplary socio-economic relation model including the presumed pathways between the six selected criteria and the criterion completion of tertiary education and the strength of the valid relation.

In line with the results of the path analysis, the validity and the strength of the relations considered in the exemplary socio-economic relation model are illustrated in Figure 3. Each straight arrow (in Figure 3) shows a valid relation between two criteria, heading from the potential factor to the condition. Dotted arrows in Figure 3 present the invalid relations according to the path analysis. Spreadsheet S4 in the Supplementary Material presents the detailed outcome of the path analysis.

The key messages gained from the path analysis (based on the exemplary socio-economic relation model) are summarized in the following bullet points and are then explained in detail.

- The criterion enrolment in tertiary education has a direct relation to the criterion completion of tertiary education.
- The criteria adolescent fertility, children involved in child labor, public expenditure on tertiary education, and children married or in union have an indirect relation to the criterion completion of tertiary education.
- The criterion enrolment in tertiary education presents the strongest relation to the criterion completion of tertiary education, followed by the criteria adolescent fertility, children involved in child labor, public expenditure on tertiary education and children married or in union.

The results of path analysis denote that the criterion completion of tertiary education is directly related to and can be predicted by the criterion enrolment in tertiary education (via P_12_ and H_12_) in the socio-economic relation model. The criteria adolescent fertility (via P_3_ and H_3_), children involved in child labor (via P_4_ and H_4_) and public expenditure on tertiary education (via P_6_ and H_6_) have direct relation to the criterion enrolment in tertiary education and thus have indirect relation to the criterion completion of tertiary education. In addition, the criterion children married or in union has indirect relation to both the criteria completion of tertiary education and enrolment in tertiary education by its direct relation to the criterion adolescent fertility (via P_1_ and H_1_). The results also show that the relation of the criterion household and ambient air pollution to the criteria enrolment in tertiary education and completion of tertiary education is not of statistical significance.

Moreover, the path analysis results indicated that the criterion enrolment in tertiary education has the strongest relation to the criterion completion of tertiary education among the selected criteria, followed by the criteria adolescent fertility, children involved in child labor, public expenditure on tertiary education and children married or in union. According to Figure 3, the criterion adolescent fertility has an indirect relation to the criterion completion of tertiary education through its direct relation to the criterion enrolment in tertiary education (via the pathways P_3_ and P_12_). The strength of the indirect relation of the criterion adolescent fertility to the criterion completion of tertiary education (path coefficient of −0.212) was derived by multiplying the path coefficients for P_3_ and P_12_. Following the same logic, the indirect relation of the criteria children involved in child labor (path coefficient of −0.190), public expenditure on tertiary education (path coefficient of 0.170) and children married or in union (path coefficient of −0.168) to the criterion completion of tertiary education were estimated. According to the magnitude of the derived path coefficients, the strength of the relation between the selected criteria and the criterion completion of tertiary education were thus compared.

Several studies provided a similar description to the key messages gained from the path analysis. For example, the criteria adolescent fertility, children involved in child labor and children married or in union were recognized as negative factors that relate to the attendance of secondary and higher levels of education (i.e., tertiary education). The criteria child marriage and adolescent fertility may limit opportunities for attending education and likely contribute to school dropout [48,49]. Delprato et al. [50] found that delaying early marriage by one year is associated with an increase of half a year of education in Sub-Saharan Africa and one third of a year of education in South West Asia. According to Presler-Marshall and Jones [51], 90% of adolescent fertility in the developing world are to girls who are married. In general, the majority (75%) of adolescent fertility are planned and associated with child marriage. These studies support that the two selected criteria adolescent fertility and children married or in union may have direct relation to the criterion enrolment of tertiary education and indirect relation to the criterion completion of tertiary education. In addition, Putnick and Bornstein [52] found a significant negative relation between child labor and enrollment of school in 30 low- and middle-income developing countries. Guarcello et al. [53] pointed out the risk that engagement in employment increases the probability of being out of school among 25 developing countries. The two studies could support the identified direct relation between the criteria children involved in child labor and enrolment of tertiary education and the potential indirect relation between the criteria children involved in child labor and completion of tertiary education (due to the school dropout). Public expenditure was identified in literature as a positive factor that contribute to tertiary education attainment. Trostel [54] found that state funding for tertiary education has significant attribution to both college enrollment and degree attainment based on 22 years of U.S. interstate data (1985–2006). Haveman and Smeeding [55] pointed out that public expenditure on tertiary education is a key factor to foster the attendance of tertiary education for the students in poor and minority neighborhoods. These two studies support that the criterion public expenditure on tertiary education has direct relation to enrolment of tertiary education and thus indirect relation to completion of tertiary education.

The results of path analysis also showed that the SCDI criteria classified into different themes can be interlinked. In the exemplary socio-economic relation model, the seven selected SCDI criteria were not classified into the same themes in the SCDI framework. The criteria enrolment in tertiary education and completion of tertiary education are classified to the theme education. The criterion adolescent fertility is categorized in the theme health, and for the criteria children married or in union and children married or in union is the theme safety. The results of the path analysis demonstrated that education topics can relate to health or safety issues and supported the interwoven linkage between socio-economic topics.

##### 4.1.2.2. Establishment of a Preliminary Social Impact Pathway

By using the results of the path analysis, a preliminary social impact pathway addressing completion of tertiary education can be illustrated based on the structure of impact pathways in environmental LCA. In environmental LCA, an impact pathway quantitatively describes the relation between inventory indicators (e.g., greenhouse gas emissions) and impacts classified into impact categories at the midpoint level (e.g., climate change) and impact categories at the endpoint level (e.g., damage to ecosystem diversity [56,57]). Impacts at the endpoint level are then linked to AoPs (e.g., ecosystem quality). The inventory results are firstly classified to a specific impact category (namely classification), and then multiplied by characterization factors which presents their relative contribution to the impact (namely characterization [56,57]). For example, the greenhouse gas emissions are classified into the midpoint impact category climate change. Per kilogram of the greenhouse gas emissions carbon dioxide, methane, and dinitrogen oxide are respectively characterized as 1, 34, and 298 kg carbon dioxide equivalent (kg CO_2_ eq.) to present their relative contribution to the impact category indicator global warming potential in kg CO_2_ eq. [58] for the midpoint impact category climate change.

**Figure 4:**
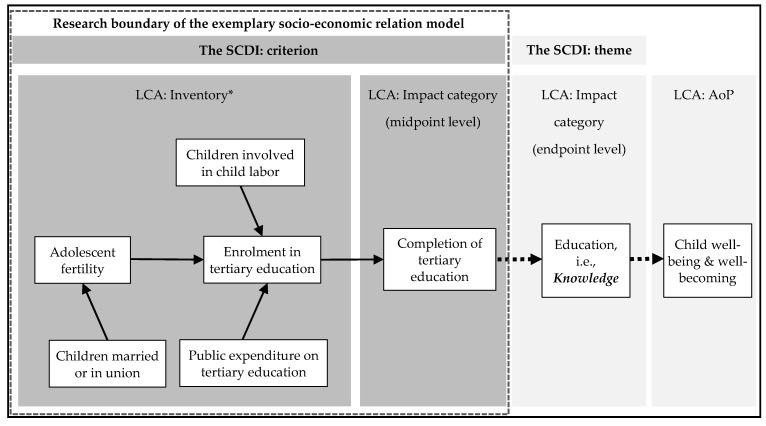
A preliminary social impact pathway addressing the criterion completion of tertiary education and its relation to the impact assessment of LCA and the SCDI framework. * Inventory denotes the selected criteria that may relate to the presumed midpoint impact category.

Taking the structure of impact pathways used in LCA as reference, completion of tertiary education, is presumed as a mid-point impact category (as shown in Figure 4). Pathways between the five criteria (i.e., enrolment in tertiary education, adolescent fertility, children involved in child labor, public expenditure on tertiary education and children married or in union) and the criterion completion of tertiary education are presented in the preliminary impact pathway. Moreover, Figure 4 maps that the presumed midpoint impact category completion of tertiary education can link to the endpoint impact category knowledge (adapted from the SCDI theme education), which links to the newly suggested area of protection for children, namely child well-being (i.e., present living state) and well-becoming (i.e., future prospects). Child well-being and well-becoming indicates how children live with the current state and how the present living state shapes children’s future prospects, connecting well-being of adults and thus societies [59]. This newly proposed area of protection closely responds to the definition of sustainable child development stated in introduction. It shall be noticed that the relation between the impact categories at midpoint and endpoint level and the area of protection (illustrated as the dotted arrows in Figure 4) is not investigated in this study. It is worthy to note that path coefficients are not identical to the characterization factors used in LCA. For example, by investigating the magnitude of path coefficients, the strength of the relation of different criteria to the presumed midpoint impact category completion of tertiary education can be compared. It does not attempt to transform the relation between different criteria and the presumed midpoint impact category into an equivalent unit.

Figure 4 also showed that the SCDI criteria could be allocated to inventory or impact level in social impact pathways (based on the structure of impact pathways in environmental LCA). This outcome is not fully identical to the proposed integration of the SCDI framework into SLCA and SOLCA (see Figure 2). In Section 4.1.1, the SCDI criteria were suggested to be as inventory indicators describing subcategories for the new stakeholder group, namely children. While we projected the examined relation between the selected SCDI criteria into an impact pathway, potential contribution of one SCDI criterion to another was found. However, it is not necessary to consider that the results of the preliminary social impact pathway conflict with the proposed integration of the SCDI framework to SLCA and SOLCA. The key reason is that one criterion could be inventory or impact according to different hypotheses of relation or in different social impact pathways. 

In brief, the exemplification demonstrates the initiative of social impact pathways based on a provision of the SCDI topics (e.g., criteria and themes) and indicators, and a quantitative evaluation of the strength of relation by path analysis. Academia specialized in social studies and development research can take the exemplification as reference to quantitatively measure the relation between socio-economic topics in general. The results of this exemplification can also be used to support governments and public bodies to design the policies regarding child development as well as sustainable development. The path analysis pointed out that enrolment of tertiary education is instrumental in fostering completion of tertiary education. Authorizations shall then consider the measures that support enrolment on tertiary education in policy making. Public expenditure is also identified as a factor that can positively contribute to enrolment of tertiary education and thus completion of tertiary education. Besides, since the criteria adolescent fertility, children married or in union, children involved in child labor were found negatively relating to the criterion completion of tertiary education, these three criteria need to be mitigated and concerned in child development or sustainable development policies for enhancing knowledge (gained from tertiary education) level of population in a country.

### 4.2. Inclusion of the SCDI into the SHDB

To overcome the missing consideration of children in the Social Hotspots Database (SHDB), the SCDI can be added as a new indicator in the SHDB to describe the degree of sustainable development for countries. For example, while using the SHDB to conduct a generic assessment, practitioners can apply the SCDI to evaluate and compare the status of sustainable child development for countries involving in a supply chain.

In addition, the SCDI can be used as a complementary assessment to the Social Hotspots Index (SHI). The SHI was designed for summarizing social risks for countries and country-specific-sectors from a whole-population-oriented perspective. For having a more complete interpretation of countries’ social conditions, the SCDI is thus recommended to be applied together with the SHI in generic assessments. It shall be noticed that the SCDI at present only provide assessment at country level.

**Figure 5:**
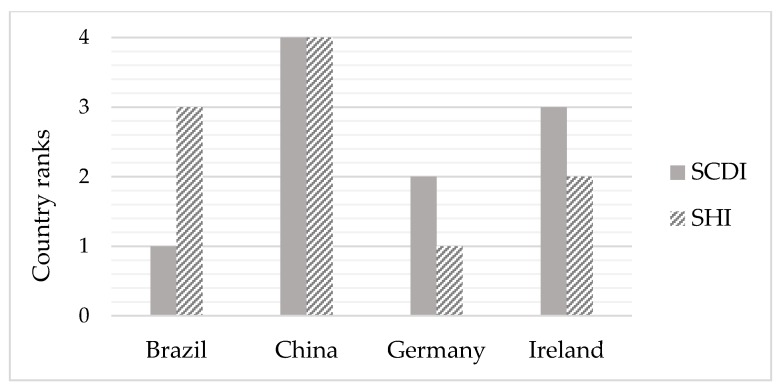
Ranking assessed by the SHI and the SCDI for the four countries involving the bamboo bike supply chain case study (1: Best, 4: Worst).

To compare the social risks of countries assessed by the SCDI and the SHI, a case study of the life cycle of a bamboo bike sold and used in Germany was conducted. Social risks evaluated for country-specific-sectors by the SHI can be used to interpret the status with regard to sustainable development for countries. According to Chang et al. [34], bamboos were assumed to be planted and processed in China; steel, aluminum, plastics and rubber components were presumed to be manufactured in Germany. Based on the German raw material situation report [60], raw material for manufacturing steel and aluminum components were mainly imported from Brazil and Ireland respectively. Hence, China, Brazil, Ireland and Germany were the four countries considered in the case study. Based on the data in SHDB, China has the highest SHI scores (210.91), followed by Brazil (112.79), Ireland (46.83) and Germany (22.08). The higher the SHI score is, the higher are social risks in a country-specific-sector. The result indicates that China and Brazil are the country assessed with the highest social risks, which could imply unfavorable status regarding sustainable development. The SCDI scores show that Brazil (0.794) has better sustainable child development status than Germany (0.793), Ireland (0.781), and China (0.724) [7]. The higher the SCDI score is, the better is the sustainable child development status for a country. To illustrate the results of the comparison, Figure 5 shows the country ranking regarding their social risks assessed by the SCDI and the SHI of the bamboo bike case study. Compared to the other considered countries, Brazil has a significant difference between the ranking assessed by the SCDI and the SHI. Different to the ranking evaluated by the SHI, Brazil shows the best ranking assessed by the SCDI among the four countries. This advantage results from having a better performance in the subthemes freshwater vulnerability and renewable energy consumption considered for the theme environmental aspects in the SCDI. Besides, only China has the same rank assessed by the SHI and the SCDI. This outcome points out that the SCDI leads to different social risks assessed for countries than the SHI by considering children as the key stakeholder group. By using the SCDI as a complementary assessment to SHI in the SHDB, organizations can screen the social risks of the countries which involve in a supply chain of a specific product to support supply chain management.

## 5. Discussion

This section presents the limitations with regard to the proposed application options of the SCDI. Research limitations are clustered into three groups: Limitations for applying the SCDI similarly to existing development indices (Section 5.1), for integrating the SCDI into SLCA, SOLCA and the SHDB as a complementary assessment (Section 5.2), and for initiating the development of quantitative social impact pathways (Section 5.3).

### 5.1. Limitations for Applying the SCDI Similarly to Existing Development Indices

First, alike the feature of all indices, the SCDI summarizes a large amount of information from the included indicators. Apart from the aggregated results, the practitioners shall also examine and show the results of individual indicators in a transparent way to avoid overlooking the potential gaps for achieving sustainable child development. Additionally, reference years of statistical data for the indicators used in the SCDI are not identical. Statistical data for most of indicators (e.g., for describing child mortality and attendance of education), are updated annually. On the other hand, some indicators (e.g., for describing mental health and renewable energy consumption) are not frequently updated. This inconsistency shall be noticed when interpreting the SCDI results, especially while monitoring the trend of sustainable child development achievement for countries.

### 5.2. Limitations for Integrating the SCDI into SLCA, SOLCA and the SHDB as a Complementary Assessment

Assessment scopes of the SCDI, SLCA, SOLCA and the SHDB are different. The SCDI evaluates the status of sustainable development for countries with regard to all the three pillars of sustainable development. Compared to SLCA, SOLCA and the SHDB, the SCDI provides a broader assessment scope by further addressing environmental aspects. Thus, different assessment scopes of the considered dimensions of sustainable development shall be noticed while applying the SCDI in SLCA and SOLCA case studies and comparing the results of the SCDI and the SHI. Additionally, it shall be noticed that the SCDI at present evaluates sustainable child development on country rather than sector/organization/product level. Results of the SCDI can be applied to reflect the generic social conditions for countries but should not be directly interpreted as social risks caused by country-specific-sectors, organizations or products.

### 5.3. Limitations for Initiating the Development of Quantitative Social Impact Pathways

The statistic technique of path analysis is based on linear regressions to examine the validity of hypotheses for the hypothesized relations. It follows the common assumptions of linear regression, e.g., data linearity, and unidirectional relation flow (e.g., no loop [21,25,61]). However, socio-economic topics are difficult to meet these presumptions of linear regression. Socio-economic topics could relate to each other within a loop. For instance, completion of tertiary education and the selected SCDI criteria could be interdependent. This interdependence brings difficulty and uncertainty in quantifying and interpreting the relation.

Reference year of statistical data for the indicators applied in the exemplary socio-economic relation model are not identical. For instance, statistical data for the indicator “adolescent fertility rate (per 1000 girls aged 15–19 years)” are updated to the year 2015. For the indicator “gross enrolment ratio in tertiary education”, the latest data for countries vary from the year 2003 to 2015. This inconsistency in statistical data can lead to uncertainty of the results of the path analysis.

Moreover, path analysis examines if a given data set fits the hypothesized relation specified in the hypothesized relation model, but it can neither prove the existence of relation, nor test compatibility of the hypothesized relation model [21,27,61]. Additionally, the comprehensiveness of considered SCDI criteria and the completeness of the proposed pathways for the considered SCDI criteria can largely influence the robustness of the results. The selected SCDI criteria and the presumed relation were used to exemplify how to quantify comparative strength of the relation between socio-economic topics. Other topics of sustainable child development can be added to extend and refine this exemplary relation model. Besides, since the indirect relation between the selected SCDI criteria were estimated by multiplying the path coefficients along the pathways, the uncertainty and statistical errors could be expanded.

In addition, it is noteworthy to discuss that the identified relation between the selected SCDI criteria does not contradict to the developed SCDI framework. The classification of the relevant topics and indicators of the SCDI was made based on the literature review in existing development studies, and it does not necessarily indicate the criteria can only relate to the criteria within the same theme. As the results of the path analysis, an education topic can relate to health and safety issues. The identified relation reflects the complex nature and interwoven linkages between socio-economic topics.

## 6. Conclusions

The Sustainable Child Development Index (SCDI) was developed in previous work to evaluate countries’ status of sustainable development by considering children as the key stakeholder and addressing topics in the context of inter-generational equity (e.g., environmental aspects). This study suggests two directions of application options to put the SCDI into practice. Both directions deal with the fact that the SCDI addresses the missing consideration of children in these sustainability assessments and databases. By putting the SCDI into practice, the SCDI can contribute to supporting decision making in development policies and enhancing existing sustainability assessments.

First, the SCDI can be used similarly to the current practice of existing development indices for comparing and tracing the status of sustainable child development on different geographic levels and population groups. The SCDI can also be expanded for including additional topics for different purposes of sustainability assessments.

Second, the SCDI framework can be integrated into existing social sustainability assessment approaches and databases to tackle the missing consideration of children and to support the development of quantitative social impact pathways. The SCDI framework can be used to complement the existing SLCA and SOLCA scheme by proposing a new stakeholder group and corresponding impact categories, subcategories and indicators connected to sustainable child development. The SCDI can be used as a reference to initiate the establishment of stakeholder-oriented indices for existing stakeholder groups in SLCA and SOLCA. In addition, by the provision of the SCDI framework and the application of path analysis, this study demonstrates how to quantify the strength of the relation between the selected SCDI criteria and the criterion completion of tertiary education and thus establishes a preliminary social impact pathway addressing completion of tertiary education. According to the path analysis, the criterion enrolment in tertiary education presents the strongest relation to the criterion completion of tertiary education, followed by the criteria adolescent fertility, children involved in child labor, public expenditure on tertiary education and children married or in union. Scholars can take the exemplification as reference to quantitatively measure the relation between socio-economic topics in general. Moreover, the SCDI can be considered in the SHDB in an effort to screen the degree of sustainable child development of countries.

The future research would focus on the implementation of the SCDI through case studies and the development of quantitative social impact pathways, and the continuous update of the SCDI framework and indicators when additional literature and statistical data regarding sustainable child development become available.

## Figures and Tables

**Table 1:** Summary of key differences between the SLCA and SOLCA framework, adapted from [10].

Method Requirement	SLCA	SOLCA
Goal	Assess social conditions and the socioeconomic performance of a product throughout its life cycle and for its stakeholders	Assess social conditions and the socioeconomic performance of an organization and its value chain and for its stakeholders
Unit of analysis	A functional unit referring to the quantified performance of a product system (e.g., a car driven for 30,000 km)	An organization and its portfolio (e.g., an organization that produces a series of cars)
Data collection	Specific data for the product assessed is expected, at least for the identified hotspots. Screening social hotspots based on generic data (country or sector level) is recommended. Collection of site-specific data is mostly done on an organization (or facility) level but not on a product level	Specific data should be used for direct activities, at least for the identified hotspots. The use of generic or extrapolated data may be used for indirect activities. Specific data are more likely to be available on organization, than on product level
Relating data to unit of analysis	Qualitative and perhaps some quantifiable data may not be expressible per unit of process or per product	Data collected for social aspects can mostly relate to the organization management and behavior in a direct way

**Table 2:** Impact categories, subcategories and indicators for the proposed stakeholder group children, adapted from the current SCDI framework [7].

Stakeholder Group	Impact Category	Subcategory	Indicator
Children	Health	Child mortality	Under-five mortality rate (probability of dying by age five per 1000 live births)
		Immunization coverage	Diphtheria tetanus toxoid and pertussis (DTP3) immunization coverage among one-year-olds (%)
		Nutrition	Percentage of infants born with low birth weight (<2500 g)
		Risk behavior	15–19 years old heavy episodic drinkers (% by country)
			Adolescent fertility rate (per 1000 girls aged 15–19 years)
		Mental health	Suicide rate (per 100,000 aged 15–29 years)
		Oral health	DMFT (decayed, missing or filled teeth) among 12-year-olds
		Health expenditure	Health expenditure, public (% of total health expenditure)
		Hazardous pollutant	Mortality rate attributed to household and ambient air pollution (per 100,000 population)
			PM2.5 air pollution, population exposed to levels exceeding WHO guideline value (% of total)
	Education	Early childhood education	Gross enrolment ratio, pre-primary, both sexes (%)
		Attendance of education	Gross enrolment ratio, primary, both sexes (%)
			Gross enrolment ratio, secondary, both sexes (%)
		Gender equality	Gross enrolment ratio, pre-primary, gender parity index
			Gross enrolment ratio, primary, gender parity index
			Gross enrolment ratio, secondary, gender parity index
			Gross enrolment ratio, tertiary, gender parity index
		Government support on education	Government expenditure on education (% of GDP)
	Safety	Violence and crime	Intentional homicide count and rate per 100,000 population
		Demographic structure	Sex ratio at birth (ratio)
	Economic status	Housing quality	Access to electricity (% of population)
		Macroeconomic situation	Youth unemployment rate (% of total labor force ages 15–24)
			Public debt (% of GDP)
	Environmental aspects	Freshwater vulnerability	Water depletion index (WDI) (ratio)
		Renewable energy consumption	Renewable energy consumption (% of total final energy consumption)

## Supplementary Materials

The following are available online at http://www.mdpi.com/1660-4601/15/7/1391/s1, Spreadsheet S1: Statistical data collected for the seven indicators considered in the linear regression models of the path analysis, Spreadsheet S2: Results of the correlation analysis of the seven indicators considered in the linear regression models of the path analysis, Spreadsheet S3: Hypotheses for potential relation between the six selected criteria and the criterion completion of tertiary education, Spreadsheet S4: Results of the three linear regression models of the path analysis.
